# Extreme Emission of N_2_O from Tropical Wetland Soil (Pantanal, South America)

**DOI:** 10.3389/fmicb.2012.00433

**Published:** 2013-01-04

**Authors:** Lars Liengaard, Lars Peter Nielsen, Niels Peter Revsbech, Anders Priemé, Bo Elberling, Alex Enrich-Prast, Michael Kühl

**Affiliations:** ^1^Department of Biology, University of CopenhagenCopenhagen, Denmark; ^2^Department of Bioscience, Aarhus UniversityAarhus, Denmark; ^3^Department of Geosciences and Natural Resource Management, University of CopenhagenCopenhagen, Denmark; ^4^Laboratory of Biogeochemistry, University Federal of Rio de JaneiroRio de Janeiro, Brazil; ^5^Plant Functional Biology and Climate Change Cluster, University of Technology SydneySydney, NSW, Australia; ^6^Singapore Centre on Environmental Life Sciences Engineering, Nanyang Technological UniversitySingapore

**Keywords:** tropical wetland, natural greenhouse gas source, microbial nitrogen cycling, nitrous oxide emission, soil oxic-anoxic transition

## Abstract

Nitrous oxide (N_2_O) is an important greenhouse gas and ozone depleter, but the global budget of N_2_O remains unbalanced. Currently, ∼25% of the global N_2_O emission is ascribed to uncultivated tropical soils, but the exact locations and controlling mechanisms are not clear. Here we present the first study of soil N_2_O emission from the Pantanal indicating that this South American wetland may be a significant natural source of N_2_O. At three sites, we repeatedly measured *in situ* fluxes of N_2_O and sampled porewater nitrate $\left({\text{NO}}_{3}^{-}\right)$ during the low water season in 2008 and 2009. In 2010, 10 sites were screened for *in situ* fluxes of N_2_O and soil ${\text{NO}}_{3}^{-}$ content. The *in situ* fluxes of N_2_O were comparable to fluxes from heavily fertilized forests or agricultural soils. An important parameter affecting N_2_O emission rate was precipitation, inducing peak emissions of >3 mmol N_2_O m^−2^ day^−1^, while the mean daily flux was 0.43 ± 0.03 mmol N_2_O m^−2^ day^−1^. Over 170 days of the drained period, we estimated non-wetted drained soil to contribute 70.0 mmol N_2_O m^−2^, while rain-induced peak events contributed 9.2 mmol N_2_O m^−2^, resulting in a total N_2_O emission of 79.2 mmol N_2_O m^−2^. At the sites of repeated sampling, the pool of porewater nitrate varied $\left(0.002-7.1\;\mu \;\text{mol}\;{\text{NO}}_{3}^{-}\;\text{g}\;\text{d}{\text{W}}^{-1}\right)$ with higher concentrations of ${\text{NO}}_{3}^{-}$ (*p* < 0.05) found in drained soil than in water-logged soil, indicating dynamic shifts between nitrification and denitrification. In the field, O_2_ penetrated the upper 60 cm of drained soil, but was depleted in response to precipitation. Upon experimental wetting the soil showed rapid O_2_ depletion followed by N_2_O accumulation and a peak emission of N_2_O $\left(2.5\text{ - 3}\text{.0}\;\;\text{m}\;\text{mol}\;{\text{N}}_{2}\text{O}\;{\text{m}}^{-2}\;\text{da}{\text{y}}^{-1}\right).$ Assuming that the observed emission of N_2_O from these wetland soils is generally representative to the Pantanal, we suggest that this undisturbed tropical wetland potentially contributes ∼1.7% to the global N_2_O emission budget, a significant single source of N_2_O.

## Introduction

The atmospheric concentration of nitrous oxide (N_2_O) is increasing at an accelerating rate with anthropogenic sources estimated to account for ∼38% of the current N_2_O emission (IPCC, 2007). This is concerning because N_2_O is a powerful greenhouse gas (IPCC, 2007) and the most important ozone depleter of the twenty-first century (Ravishankara et al., 2009). Modeling of the future global climate is dependent on our understanding of the mechanisms that control the atmospheric concentration of greenhouse gases (CO_2_, CH_4_, and N_2_O) and our ability to obtain an accurate budget of anthropogenic and natural sources and sinks. Microbial processes play a major role in the global cycling of carbon and nitrogen (e.g., Gruber and Galloway, 2008) and while the cycling of both is closely linked (e.g., Schlesinger, 2010) most focus has been on carbon. Both CH_4_ and N_2_O are primarily biogenic (IPCC, 2007) and methanogens, nitrifiers, and denitrifiers are key players in regulating the global sources and sinks of CH_4_ and N_2_O. This underscores the need to better understand how environmental parameters and microorganisms interact to become sources or sinks of these greenhouse gases on a larger scale. In this study the focus is on tropical wetlands soils and we present *in situ* evidence that the world’s largest freshwater wetland (Pantanal, Brazil) acts as significant as a source of N_2_O.

Although our knowledge of sources and sinks of N_2_O in different environments is increasing, the global N_2_O budget remains unbalanced (Smith, 1997; IPCC, 2007). Currently, ∼25% of the global N_2_O emission is ascribed to uncultivated tropical soils, but the exact locations and controlling mechanisms, including the role of microbial processes, are not clear (D’Amelio et al., 2009). Furthermore, a recent study of the tropospheric distribution and variability of N_2_O demonstrated that global sources of N_2_O are concentrated in the tropics and suggests that South America has an up to five times higher emission of N_2_O than expected (Kort et al., 2011).

Several studies indicate that tropical forest ecosystems simultaneously accumulate, recycle, and loose nitrogen in far larger quantities than temperate ecosystems (Martinelli et al., 1999; Matson et al., 1999; Hedin et al., 2009). In spite of being rich in nitrogen, this recycling may include high bacterial N_2_-fixation activity, a nitrogen paradox (Hedin et al., 2009) enabling tropical forest ecosystems to sustain large gaseous nitrogen losses (Houlton et al., 2006). Half of the world’s wetland areas are found in the tropics (Neue et al., 1997); and if the nitrogen paradox is similarly applicable in these ecosystems, tropical wetlands may have a major and yet unresolved role in the global budget of atmospheric N_2_O.

In both natural and impacted environments, the same microbial processes are responsible for the production of N_2_O, i.e., nitrification $\left({\text{NH}}_{4}^{+}\to {\text{O}}_{2}\to {\text{NO}}_{3}^{-}\right)$ and denitrification $\left({\text{NO}}_{3}^{-}\to {\text{NO}}_{2}^{-}\to \text{NO}\to {\text{NO}}_{2}^{-}\to {\text{N}}_{2}\right)$. Nitrifiers in soil can be stimulated to release N_2_O at low O_2_ availability (∼5% air sat.; Bollmann and Conrad, 1998), soil moisture content of up to ∼60% WFPS (Bateman and Baggs, 2005) and low pH (Mørkved et al., 2007). Denitrifying microorganisms can be stimulated to release N_2_O by sudden onset of anoxia (Morley et al., 2008), high concentrations of ${\text{NO}}_{3}^{-}$ (Blackmer and Bremner, 1978; Blicher-Mathiesen and Hoffmann, 1999), and low pH (Simek and Cooper, 2002). Both nitrification and denitrification can occur simultaneously in complex soil microsites with different access to O_2_. This makes it difficult to associate a measured soil N_2_O emission with a specific microbial process (Stevens et al., 1997). However, denitrification is often considered the main N_2_O producing process in soils (Dobbie et al., 1999; Abbasi and Adams, 2000).

The continuous cycle of flooding and draining of wetlands affects important environmental soil parameters such as their O_2_ content, pH, and redox potential and thereby modulates the biogeochemical processes involved in production and emission of N_2_O (Baldwin and Mitchell, 2000). This hydrological pulse effect is well known in systems influenced by anthropogenic input of nitrogen, where hot spots or hot moments (McClain et al., 2003) of N_2_O emission are induced by temporal or spatial oxic-anoxic transitions in for example riparian marshes (Hernandez and Mitsch, 2006), agricultural soil (Markfoged et al., 2011), and mangrove sediment (Allen et al., 2007). Similar flooding effects in tropical freshwater systems are much less explored.

Here we present the first study of the *in situ* fluxes of N_2_O, the dynamics of soil nitrogen pools and soil O_2_ content in Pantanal wetland soils at different times and water status. Three sites were sampled repeatedly over a period of 23–42 days in the beginning of the low water season in 2008 and 2009. Additionally, in the end of the low water season in 2010 10 sites were screened for *in situ* surface flux of N_2_O and soil ${\text{NO}}_{3}^{-}$ content.

## Materials and Methods

### Study sites

**Figure 1:**
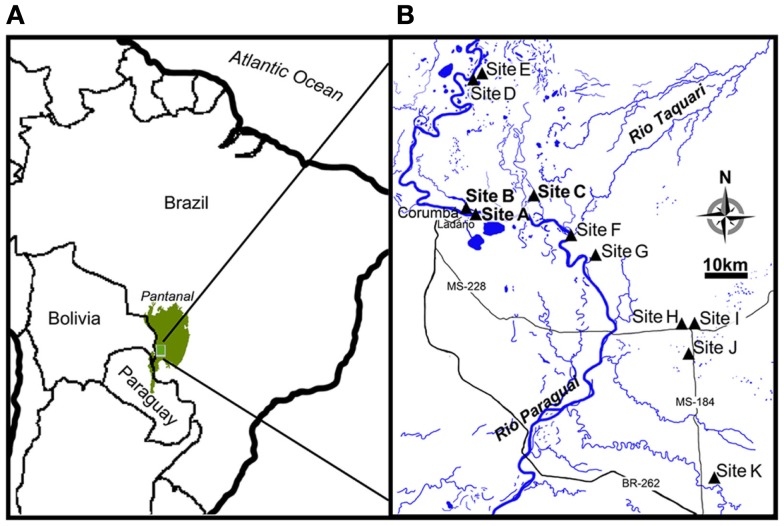
**Location of Pantanal and map insert of the study site**. **(A)** Pantanal is shown as green area in the center of South America. **(B)** The course of Rio Paraguai flowing from north to south in the Pantanal and locations of the sampling sites (site A–K).

**Figure 2:**
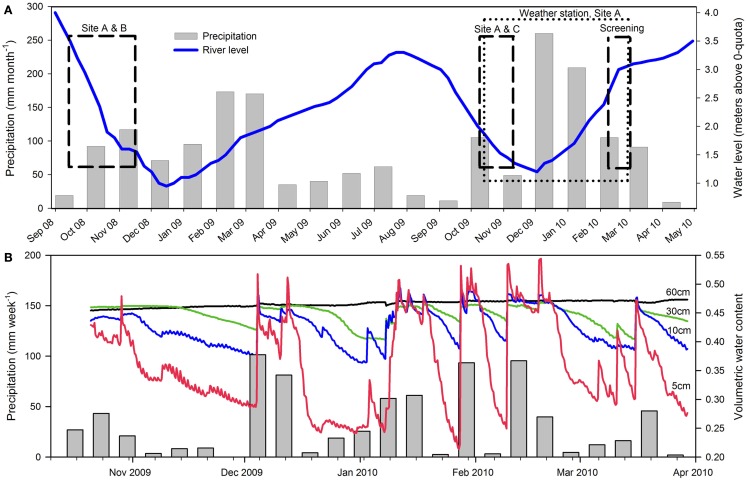
**Precipitation and the water level in Rio Paraguai during 2008–2010**. **(A)** Monthly precipitation at Corumbá Airport is shown as gray bars (source: www.inmet.gov.br). Daily water level of Rio Paraguai at Ladário Naval Station is shown as blue line (source: www.cprm.gov.br). The period of each field campaign in 2008, 2009, and 2010 is indicated with black dashed boxes, while the dotted box indicates the period of precipitation, soil moisture, and soil temperature measurements at site A. **(B)** Precipitation and soil moisture content in 2009–2010 at site A, Level 2. Weekly precipitation is indicated as gray bars. Soil moisture at different depths below the soil surface is shown as lines; 5 cm (red), 10 cm (blue), 30 cm (green), and 60 cm (black) below the soil surface.

The Pantanal, a pristine tropical wetland in central South America, is shaped by the deposition of sediments into a tectonic depression in the Upper Paraguay River Basin, which formed during the last Andean compressive event (∼2.5 Ma; Assine and Soares, 2004). The Pantanal supports a lush vegetation of floating and emersed herbs and is dominated by an annual flooding and precipitation cycle, alternately inundating and draining ∼140,000 km^2^ of soil (Swarts, 2000), thus representing the world’s largest wetland (Figure 1A). Aquatic macrophytes and herbaceous plants colonize the entire gradient from permanently dry to permanently wet conditions. In the aquatic-terrestrial transition zone, the herbaceous plant communities die off when the water floods the area annually; this is often followed by an anoxic event in the river and flood water due to the massive decomposition of vegetation (Hamilton et al., 1997; Calheiros et al., 2000). During the flooded season, aquatic macrophytes like *Eichhornia crassipes* and *Salvinia auriculata* dominate the ecosystem, but decompose when left on the draining wetland soil as the water level decreases (Junk et al., 2006). The flooding pulse thus leads to a regular set-back of community development maintaining the system in an immature, but highly productive stage (Junk and Wantzen, 2004). The annual flooding cycle is driven by a distinct dry/wet season in the ∼500,000 km^2^ watershed, but due to a North-South slope of only 2–3 cm km^−1^, there is a lag period of up to several months between precipitation in the watershed and the flooding of the Pantanal (Junk et al., 2006). This explains why the Pantanal receives the highest precipitation in the low water (drained) season (Figure 2A).

During three field campaigns in 2008 (42 days), 2009 (23 days), and 2010 (14 days), we investigated the *in situ* flux of N_2_O and the soil nitrogen pools from wetland soil at representative sites, near the retreating edge of typical water bodies with temporary connection to the main water courses in the Pantanal (Figure 1B). In 2008 and 2009 a main site (site A: 19°01.16′S; 57°32.99′W) was chosen for repeated sampling. An additional site was chosen for repeated sampling in 2008 only (site B: 19°0.61′S; 57°33.51′W) and in 2009 only (site C: 18°59.28′S; 57°25.17′W). In 2010, 10 sites (A, B, D–K) representing different soil and habitat types were screened (Figure 1B; Table 1). All sampling was conducted during the drained season: In 2008 and 2009 sampling was conducted during the period of retreating water, while the screening in 2010 was conducted during the period of rising water level (Figure 2A). Precipitation and soil water content was measured for 170 days of the drained season 2009–2010 (Figure 2B).

**Table 1:** **Screening of *in situ* N_2_O flux, pH, and porewater ${\text{NO}}_{3}^{-}$ in 2010 at 10 sites**.

Location	Position (WGS 84)	Level	*In situ* N_2_O flux (mmol N_2_O m^−2^ day^−1^)	pH	${\text{NO}}_{3}^{-}$ $\left(\mu \text{mol}\;{\text{NO}}_{3}^{-}\;\text{c}{\text{m}}^{-3}\text{soil}\right)$
Site A	19°01.16′S; 57°32.99′W	1	0.41 ± 0.05	5.32	0.19
		2	0.31 ± 0.02	4.96	0.91
		3	0.61 ± 0.29	4.43	2.69
Site B	19°00.61′S; 57°33.51′W	1	0.21 ± 0.03	4.37	0.16
		2	0.48 ± 0.12	4.13	3.45
Site D	18°43.56′S; 57°32.12′W	−	0.27 ± 0.02	4.50 ± 0.12	0.07 ± 0.04
Site E	18°44.08′S; 57°32.38′W	1	0.77 ± 0.18	5.08 ± 0.11	0.55 ± 0.08
		2	1.08 ± 0.17	4.75 ± 0.06	5.38 ± 2.94
Site F	19°04.26′S 57°20.08′W	1	0.22 ± 0.01	6.90 ± 0.06	0.03 ± 0.00
		2	0.41 ± 0.02	6.25 ± 0.03	0.05 ± 0.03
Site G	19°06.03′S; 57°16.85′W	−	0.18 ± 0.04	5.33 ± 0.06	0.79 ± 0.12
Site H	19°15.15′S; 57°04.83′W	−	0.20 ± 0.02	5.98 ± 0.10	0.02 ± 0.01
Site I	19°15.03′S; 57°04.04′W	−	0.45 ± 0.09	5.18 ± 0.08	0.07 ± 0.02
Site J	19°18.53′S; 57°03.29′W	−	0.09 ± 0.01	7.48 ± 0.08	0.29 ± 0.11
Site K	19°34.50′S; 57°01.22′W	1	0.30 ± 0.01	−	0.06
		2	0.30 ± 0.01	−	0.31

*Mean ± SE (*n* = 5)*.

Measurements and sampling were carried out along 10–25 m long transects ranging from the retreating water edge to ∼1 m above the initial water edge at sites A, B, and C. At the beginning of each field campaign in 2008 and 2009, the elevated end of transects had been drained for ∼1 month, while the lowermost end was initially water-logged, but drained during the field campaign. Three levels were selected along each transect: Level 1 at the lowest end, Level 2 in the center, and Level 3 at the highest end, thus representing both an increase in elevation and a gradual decrease in soil moisture content from Level 1 to Level 3. Due to an excessive amount of rain on site C during the 2009 field campaign, this site was water-logged during all measurements, while early flooding of site C in 2010 left it inaccessible.

### *In situ* flux measurements of N_2_O

At each site, the *in situ* flux of N_2_O was measured at the two lowest levels (Level 1 and 2) along the transect. Flux chambers (*n* = 5 at each level) made of PVC tubes (∅ = 24 cm, height = 20 cm) were inserted ∼15 cm into the soil. If any litter layer was present on the soil surface prior to inserting the chamber, it was replaced on the soil surface inside the chamber. *In situ* flux measurements were performed every 2–14 days by placing a lid on the tube and measuring the N_2_O concentration for 30 min in each chamber with a photo-acoustic gas monitor (INNOVA 1312, LumaSense, Inc., Ballerup, Denmark). Flux chambers were lined with reflective material on the outside and were shaded during measurements to minimize temperature variations. The closed-chamber technique is known to create a bias by altering the diffusion gradient between soil and chamber headspace (Anthony et al., 1995). However, several studies have shown that this bias can be overcome by applying a non-linear regression method to describe the gas exchange (e.g., Kroon et al., 2008; Forbrich et al., 2010). In our case, the flux in each chamber at *t* = 0 was estimated by fitting the partial pressure increase to a three-parameter exponential function [pp*_t_* = pp_0_ + a(1−*e*^−b*t*^)] in Sigmaplot (Systat Software, Inc., Chicago, IL, USA), where pp*_t_* is the partial pressure of the measured gas at time *t*, pp_0_ is the initial partial pressure in the closed-chamber, *t* is time, and a and b are constants. Integrated emissions of N_2_O were calculated for each level at each site, assuming linearity between subsequent measurements.

During the 2010 screening, the sites had to the best of our knowledge not received precipitation in the preceding days, and the *in situ* measurements are thus assumed to represent drained soil fluxes.

### Soil parameters

#### Porewater ${\text{NO}}_{3}^{-}$

Whole soil cores (∅ = 5.5 cm, length = 15 cm) were collected at all levels (*n* = 3 at each level) on every sample occasion at site A, site B, and site C (2008: three times at site A, three times at site B, 2009: five times at site A, four times at site C) and soil porewater immediately extracted *in situ* by inserting 0.2 μm Rhizon filters (Rhizosphere Research Products, Wageningen, Netherlands) into the side of the whole soil cores at 1.5, 3.5, and 6.5 cm below the soil surface. Samples of ∼0.5 ml of porewater were extracted at each depth by suction with a 60 ml syringe. Additional water samples were taken from rivers and water bodies and filtered (0.2 μm filter, Sartorius AG, Göttingen, Germany). Extracted porewater samples and water samples were immediately transferred to 1.5 ml tubes and stored on ice until return to the field laboratory, where they were stored at −20°C until further analysis. Nitrate analysis (sample size 5 μl) was performed using the vanadium chloride reduction method (Braman and Hendrix, 1989) in combination with a chemoluminescence detector (CLD 86, Eco Physics AG, Dürnten, Switzerland), calibrated (*r*^2^ = 1.00) at nine different concentrations of ${\text{NO}}_{3}^{-}$ (0, 25, 50, 75, 150, 300, 500, 750, 1500 μM). After extraction of soil porewater, the soil cores were sliced and the soil water content and soil dry weight was determined by weighing the soil slices before and after drying at ∼60°C for 48 h. Data from each soil ${\text{NO}}_{3}^{-}$ profile was averaged (arithmetic mean) over the upper 6.5 cm soil column.

During the third field campaign (2010), soil ${\text{NO}}_{3}^{-}$ was measured in a field laboratory with a ${\text{NO}}_{3}^{-}$ biosensor (Unisense A/S, Aarhus, Denmark), calibrated (*r*^2^ = 0.99) at six different concentrations of ${\text{NO}}_{3}^{-}$ (0, 20, 40, 60, 260, 660 μM). Soil samples (*n* = 1−3) from the mixed upper ∼5 cm soil were collected by inserting a 50 ml screw cap centrifuge tube (Sarstedt AG, Nürmbrecht, Germany) directly into the soil. Soil samples were refrigerated up to 48 h until ${\text{NO}}_{3}^{-}$ was measured in a 1% (wt/wt) NaCl solution in the field laboratory. The reason for adding NaCl was primarily a higher stability of the ${\text{NO}}_{3}^{-}$ biosensor reading in a saline solution.

Means of porewater ${\text{NO}}_{3}^{-}$ at site A, B, and C were analyzed with a GLM (two-way-ANOVA) with time and levels as factors. A Tukey’s test was run for comparisons among means. Results were tested at a significance level of 95%. Analyses were performed using SAS 9.2 (SAS Institute, Inc., Cary, NC, USA).

#### Distribution of O_2_ in soil

In 2009, the depth distribution of O_2_ concentration was measured at site A and C at Level 2 (center of transect) with custom-built fiber-optic O_2_ optodes (∅ = 2 mm; Rickelt et al., under review). The optical fibers were calibrated in an O_2_-free solution (0.2 M ascorbate, pH 12) and in water equilibrated with atmospheric air prior to installation in the soil at 13 fixed depths (2.5, 5, 10, 15, and 20–100 cm with 10 cm intervals). The optodes were connected to a four-channel fiber-optic O_2_ detector system (OXY-4, Presens GmbH, Regensburg, Germany) at each visit to the sites (site A: *n* = 22, site C, *n* = 9).

#### Soil moisture and precipitation

Precipitation, soil temperature, and seasonal changes in volumetric soil water content were measured for a period of 170 days (2009–2010) at site A, Level 2. The volumetric soil water content was logged using soil moisture sensors (Theta-Probes ML2x, Delta-T Devices, Ltd., Cambridge, UK) installed in four depths; 5, 10, 30, and 60 cm below the soil surface in one profile. Each probe was calibrated in the laboratory using depth-specific soil samples from the site. Precipitation was measured using a “Tipping Bucket” rain gage. Soil temperature and precipitation sensors were logged at 10 min intervals, while the Theta-probes were logged every 6 h (CR10X Datalogger, Campbell Scientific, Ltd., Loughborough, UK).

### N_2_O dynamics in soil wetted either experimentally or by natural precipitation

Dry soil cores (∅ = 5.5 cm, length = 15 cm) for experimental wetting were collected from level 3 at site A and site B. Further soil cores were collected immediately after a natural, moderate precipitation event of short duration (<15 min). Optical O_2_ microoptodes were constructed according to Klimant et al. (1995) and mounted in hypodermic needles and connected to a fiber-optic O_2_ meter (Microx TX3, Presens GmbH, Regensburg, Germany). Electrochemical N_2_O microsensors were constructed according to Andersen et al. (2001) with a fortified outer casing to avoid breaking the sensor in the coarse wetland soil (Markfoged et al., 2011) and connected to a picoammeter (PA2000, Unisense A/S, Aarhus, Denmark). Both types of sensors had tip diameters of ∼100 μm and a detection limit of ∼5 Pa. The O_2_ sensors were linearly calibrated from a two-point calibration in O_2_-free solution (20% ascorbic acid, pH 11), and in water equilibrated with atmospheric air. The N_2_O sensors were linearly calibrated from a two-point calibration in a 0 and 1% solution of N_2_O produced by mixing 0.5 ml of saturated N_2_O water into 49.5 ml water. The sensors were mounted on a motorized micromanipulator and both sensor position and data collection were controlled by a PC running SensorTrace Pro software (Unisense A/S, Denmark). Retrieved dry soil cores were wetted from below with aerated river water and concentration profiles (*n* = 20–30) of O_2_ and N_2_O were obtained over the following 56–72 h. Additional profile measurements were done in a soil core retrieved after wetting by a natural rain event. The N_2_O flux, *J*, was calculated from the concentration gradient in the water layer on the soil surface using Fick’s first law (*J* = −D δC/δx), where D is the N_2_O diffusivity in water at experimental temperature (2.41 × 10^−5^ cm^2^ s^−1^), C is the N_2_O concentration in μmol l^−1^ calculated from the measured partial pressure and the experimental temperature according to Weiss and Price (1980), and *x* is the vertical distance in cm.

## Results

### *In situ* flux measurements of N_2_O

**Figure 3:**
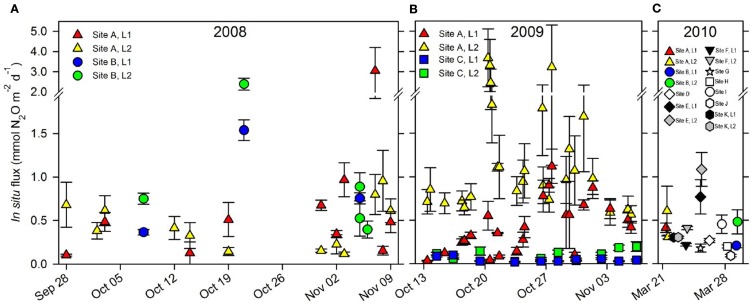
***In situ* flux of N_2_O (mean ± S. E., *n* = 5) from all sites 2008–2010**. **(A)** *In situ* flux of N_2_O in 2008 from site A, Level 1 (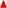) and Level 2 (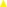) and from site B, Level 1 (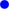) and Level 2 (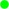). **(B)** *In situ* flux of N_2_O in 2009 from site A, Level 1 (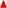) and Level 2 (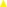) and from site C, Level 1 (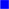) and Level 2 (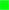). **(C)** *In situ* flux of N_2_O in 2010 from site A, B, and site D–K. Note scale break on *y*-axis.

The *in situ* flux of N_2_O at the sites of repeated sampling varied considerably over time in both 2008 (Figure 3A) and 2009 (Figure 3B) the mean daily flux of N_2_O varying between 0.04 and 1.37 mmol N_2_O m^−2^ day^−1^ at site C, Level 1 and site A, Level 2 respectively (Table 2). *In situ* fluxes of N_2_O during the 2010 screening (Figure 3C) varied between 0.09 mmol N_2_O m^−2^ day^−1^ (site J) and 1.08 mmol N_2_O m^−2^ day^−1^ (site E; Table 1).

**Table 2:** **Integrated flux and mean daily flux of N_2_O at each Level at the sites of repeated sampling**.

Site	Level	days	Integrated N_2_O flux mmol N_2_O m^−2^	Mean N_2_O flux mmol N_2_O m^−2^ day^−1^
A	1^†^	42	22.0 ± 5.6	0.52 ± 0.13
	2^†^	42	14.0 ± 3.4	0.33 ± 0.08
	1^‡^	23	12.9 ± 1.7	0.55 ± 0.07
	2^‡^	23	32.0 ± 5.0	1.37 ± 0.21
B	1	29	17.8 ± 1.8	0.64 ± 0.06
	2	29	26.7 ± 4.0	0.92 ± 0.14
C	1	23	1.0 ± 0.1	0.04 ± 0.00
	2	23	2.1 ± 0.4	0.09 ± 0.02

*Mean ± SE (*n* = 5)*.

*N.B. site C was completely water-logged during the entire field campaign*.

*^†^2008, ^‡^2009*.

**Figure 4:**
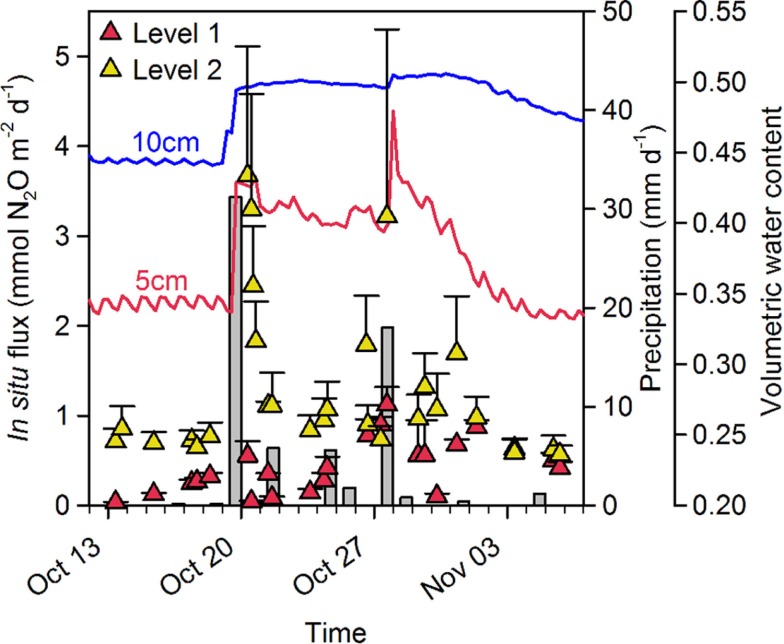
***In situ* N_2_O flux, precipitation and soil moisture content at site A in 2009**. Precipitation (gray bars: daily precipitation) caused a sudden increase in soil water content (5 cm: red line, 10 cm: blue line) in the top 10 cm soil layer and triggered an increase in the *in situ* flux of N_2_O [Mean + SE (*n* = 5)] at Level 1 (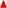) and Level 2 (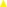).

Peak events of *in situ* N_2_O flux were apparently closely associated with sudden and heavy precipitation causing increasing soil water content in the upper 10 cm soil layer which was followed by an increased flux of N_2_O measureable 6–12 h later (Figure 4).

### Soil parameters

#### Porewater ${\text{NO}}_{3}^{-}$

**Figure 5:**
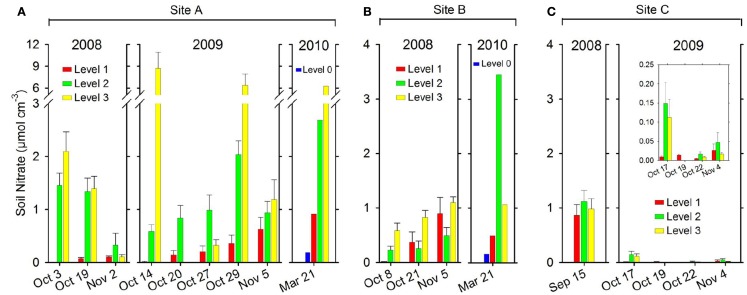
**Soil porewater ${\text{NO}}_{3}^{-}$ per soil volume (μmol N cm^−3^ soil) measured along a transect from Level 1 to Level 3 at each site**. **(A)** site A, **(B)** site B, and **(C)** site C and site D–K (one or two levels only) in 2008, 2009, and 2010. Mean + SE (*n* = 9). At site A and site B a Level 0 was introduced in 2010 because the water level was much lower by the end of the drained season. Level 0 was situated close to the water edge and therefore equivalent to Level 1 with regard to distance to the water edge in 2008 and 2009. Please note scale break on *y*-axis on (A and D) and insert figure with different scale on **(C)**.

At site A (2008) the porewater ${\text{NO}}_{3}^{-}$ content increased significantly (*p* < 0.05), going from wet soil at Level 1 to drained soil at Level 3 (Figure 5A). In addition, over time the porewater ${\text{NO}}_{3}^{-}$ content increased significantly (*p* < 0.05) at Level 1 as the soil drained, while a significant decrease (*p* < 0.05) was observed at Level 2 and 3 (Figure 5A). In 2009 the porewater ${\text{NO}}_{3}^{-}$ content at site A similarly increased significantly (*p* < 0.05) from wet soil at Level 1 to drained soil at Level 3 (Figure 5A). In addition, a significant increase (*p* < 0.05) over time in porewater ${\text{NO}}_{3}^{-}$ content was observed at Level 1. The same trend was observed between levels at site A in 2010, with porewater ${\text{NO}}_{3}^{-}$ content increasing from Level 0 to Level 3 (Figure 5A).

At site B in 2008 (Figure 5B) the porewater ${\text{NO}}_{3}^{-}$ content at Level 3 was significantly higher (*p* < 0.05) than at Level 1 and Level 2. In addition, a significant increase (*p* < 0.05) over time in porewater ${\text{NO}}_{3}^{-}$ content was observed at Level 1 and Level 3 (Figure 5B). The same trend was observed between levels at site B in 2010, with porewater ${\text{NO}}_{3}^{-}$ content increasing from Level 0 to Level 2 (Figure 5B).

In 2008 site C (Figure 5C) soil samples (*n* = 3) collected on a single occasion showed a soil ${\text{NO}}_{3}^{-}$ content of $0.73\;\pm \;0.09\;\mu \text{mol}\;{\text{NO}}_{3}^{-}\text{g}\;\text{d}{\text{W}}^{-1}$. However, in 2009 (Figure 5C) the overall porewater ${\text{NO}}_{3}^{-}$ content at site C was much lower than at site A or B, presumably due to the water-logging of the soil. Comparing between levels at site C in 2009 (Figure 5C, small insert) the porewater ${\text{NO}}_{3}^{-}$ content at Level 1 was significantly lower (*p* < 0.05) than at Level 2, but not compared to Level 3.

The soil characteristics from the 2010 screening of 10 sites are shown in Table 1. Soil ${\text{NO}}_{3}^{-}$ content varied between 0.06 μmol cm^−3^ soil (site K) and 5.38 μmol cm^−3^ soil (site E).

**Figure 6:**
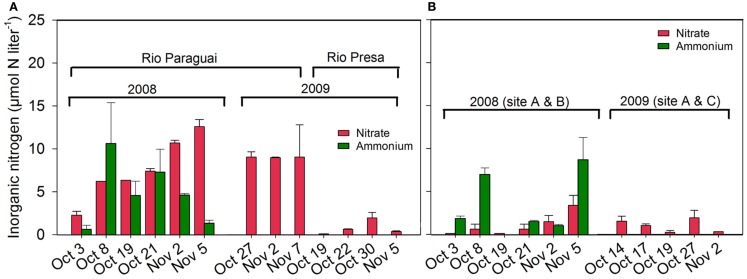
**Inorganic nitrogen (${\text{NH}}_{4}^{+}$ and ${\text{NO}}_{3}^{-}$) in the water phase in (A) Rio Paraguai and Rio Presa and in (B) water bodies near Site A, B, and C**.

The ${\text{NH}}_{4}^{+}$ concentration in river waters ranged between 0.6 ± 0.5 and $10.6\;\pm \;4.8\;\mu \text{mol}\;{\text{NH}}_{4}^{+}\;{\text{l}}^{-1}$ (Figure 6A) and ranged between 1.0 ± 0.1 and $8.7\;\pm \;2.6\mu \text{mol}\;{\text{NH}}_{4}^{+}\;{\text{l}}^{-1}$ in the other water bodies (Figure 6B). The ${\text{NO}}_{3}^{-}$ concentration in rivers ranged between 0.1 and $12.6\pm 0.8\mu \text{mol}\;{\text{NO}}_{3}^{-}\;{\text{l}}^{-1}$ (Figure 6A) and ranged between 0.1 and $3.4\pm 1.2\mu \text{mol}\;{\text{NO}}_{3}^{-}\;{\text{l}}^{-1}$ in the other investigated water bodies (Figure 6B).

#### Depth distribution of O_2_ in soil

**Figure 7:**
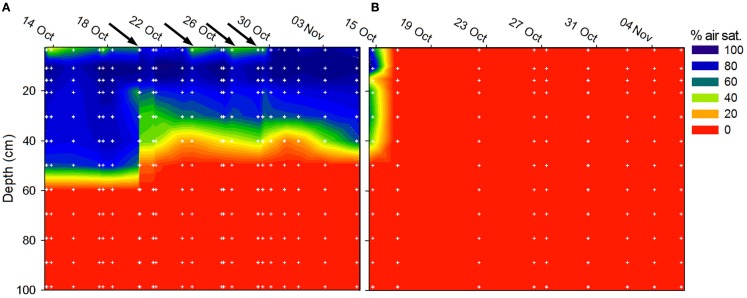
**Soil O_2_ concentration at (A) site A, Level 2, and (B) site C, Level 2 during field work in 2009**. The O_2_ concentration maps were compiled from individual oxygen profiles (*n* = 22 at site A, *n* = 9 at site C) composed of measurements at 12 depth (white dots). The black arrows on the time scale in subfig **(A)** indicate precipitation events as measured at site A, Level 2.

At site A, Level 2, O_2_ penetrated to a depth of ∼60 cm (37–97% air sat.) but fluctuated throughout the field campaign in response to precipitation (Figure 7A). At site C, Level 2, O_2_ was not detected in the soil at Level 2 except the first measurement (Figure 7B) in consistency with the soil being water-logged during the field campaign.

#### Soil moisture and precipitation

Precipitation measured at Level 2 at site A resulted in an increase in soil moisture. This was most pronounced near the soil surface (5 cm) and less in the deeper soil layers. A subsequent decrease in soil moisture occurred after each rain event, most rapidly at the surface due to evaporation and drainage (Figure 2B).

### Microsensor measurements in wetted soils

**Figure 8:**
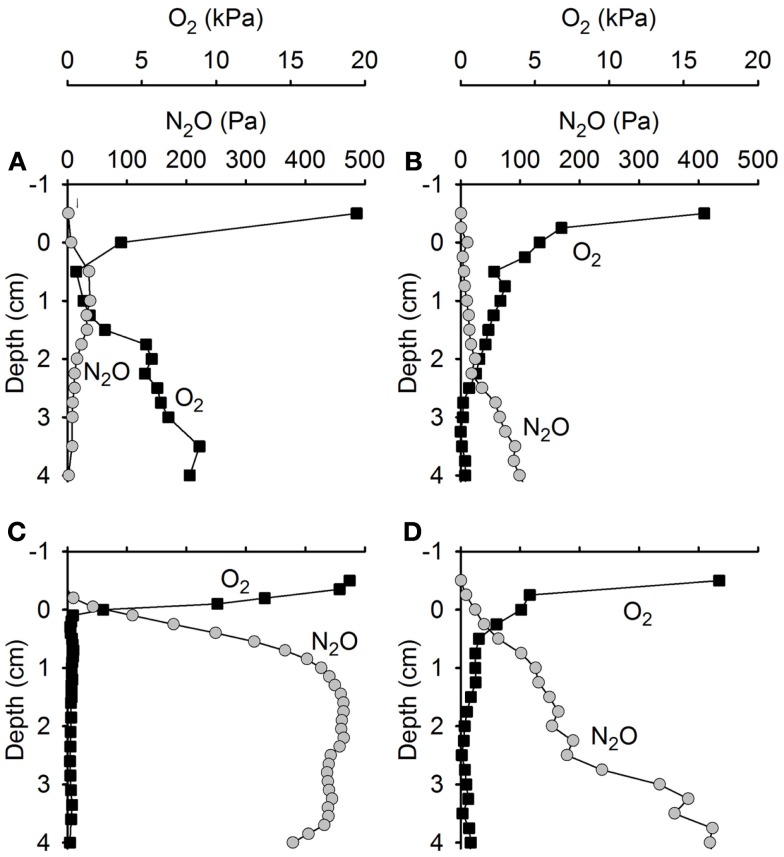
**Concentration profiles of N_2_O and O_2_ in soil cores from (A,C) site A and (B,D) site B experimentally wetted to simulate precipitation**. N_2_O and O_2_ concentrations change rapidly after wetting of the soil. **(A,B)** Four hours after wetting. **(C,D)** Ten hours after wetting.

**Figure 9:**
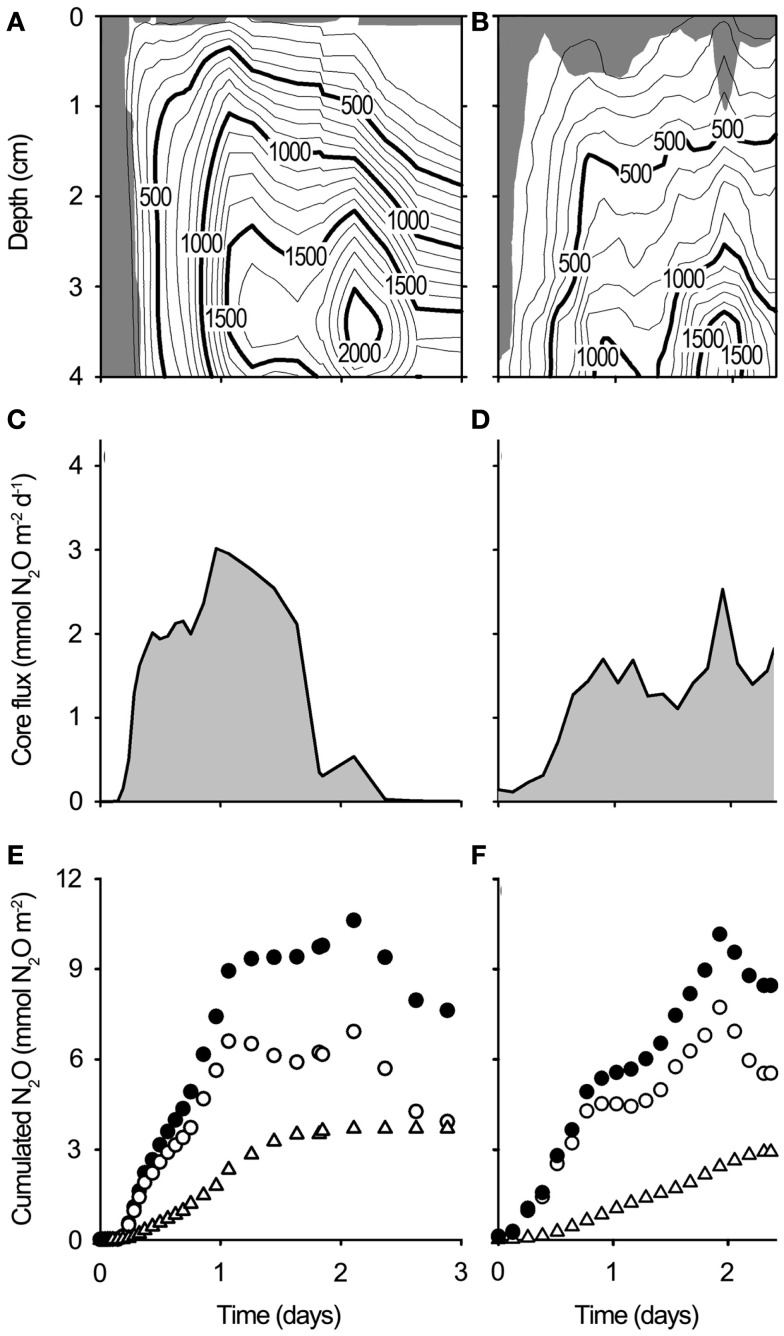
**N_2_O dynamics in whole soil cores from Site A and Site B experimentally re-wetted to simulate precipitation**. **(A,B)** Contour plots showing the N_2_O concentration across time and depth in re-wetted soil cores [**(A)**: Site A, **(B)**: Site B]. Units are pressure equivalents (Pa). The soil surface is 0 cm. The shaded area indicates the oxygenated zone (>1 kPa O_2_). **(C,D)** N_2_O emission from re-wetted soil cores [**(C):** Site A, **(D)**: Site B] as calculated from the N_2_O gradient in the water layer above the soil. **(E,F)**, Cumulated N_2_O net production (●), cumulated N_2_O emission (∆), and cumulated soil N_2_O (ം) in re-wetted soil cores [**(E)**: Site A, **(F)**: Site (B)].

Upon wetting of drained soil cores collected from Level 3 at site A and site B, porewater O_2_ depletion occurred within a few hours (Figures 8A–D), followed by a rapid accumulation of N_2_O. The accumulation of N_2_O within the soil persisted for 2–3 days (Figures 9A,B). During this period, N_2_O diffused into the overlying air causing peak emissions of 3.02 mmol N_2_O m^−2^ day^−1^ from the Site A core (Figure 9C) and 2.53 mmol N_2_O m^−2^ day^−1^ from the Site B core (Figure 9D), i.e., peak events similar in size and timing to the emission peaks found by *in situ* flux measurements.

The integrated emission from the experimentally flooded soil of 3.7 mmol N_2_O m^−2^ over 3 days (soil core from site A) and 2.92 mmol N_2_O m^−2^ over 2.3 days (soil core from site B) represented only a small fraction (35–38%) of the net production of N_2_O in the upper 4 cm of the soil matrix, the difference being consumed within the water-logged soil 2–3 days after the wetting event (Figures 9E,F).

## Discussion

Our study represents the first study of soil N_2_O emission and the dynamics of the porewater nitrate in the Pantanal and revealed a large and hitherto unknown source of N_2_O in the largest wetland of the world. In this discussion, we relate our observed *in situ* N_2_O fluxes to the soil porewater nitrate and soil moisture and precipitation data and compare these results with those found for other tropical systems. Thereafter, we discuss our findings of N_2_O production, accumulation, and emission in experimentally wetted soil cores in relation to studies of parameters influencing the reduction of N_2_O in soil. Finally we speculate on how the observed emission of N_2_O could be supported in a natural system like the Pantanal and how this might link into the cycling of carbon.

### *In situ* flux of N_2_O

The measured fluxes of N_2_O from Pantanal wetland soils had a high temporal variability (Figure 3) and were generally high (Tables 1 and 2). The average N_2_O emission from the Pantanal wetland soils was 10–390 times higher when compared to other unfertilized tropical systems (Matson and Vitousek, 1987), and 4–6 times higher when compared to fertilizer-induced N_2_O emission peaks in tropical forest soils (Hall and Matson, 1999). The fluxes of N_2_O from the Pantanal wetland soil were thus comparable to fluxes reported from heavily fertilized forest or agricultural soils receiving regular inputs of nitrogen (e.g., Hall and Matson, 1999; Ruser et al., 2006). In soil fertilized with high ${\text{NO}}_{3}^{-}$ concentrations the primary end product of denitrification upon wetting is often N_2_O (Ruser et al., 2006), and although not fertilized, the drained Pantanal wetland soils similarly contained high concentrations of ${\text{NO}}_{3}^{-}.$ The high soil ${\text{NO}}_{3}^{-}$ content thus explains the high emission of N_2_O from the drained Pantanal wetland soils.

Disturbance of tropical soil (e.g., by conversion from forest to pasture or cyclic flooding) has also been shown to increase the emission of N_2_O (Keller et al., 1993; Kern et al., 1996; Veldkamp et al., 1999). In the Pantanal the development of the plant community is continuously disturbed by the alternating flooding and draining of the soil converting the almost exclusively terrestric system to an almost exclusively aquatic system (Junk and Wantzen, 2004). Such disturbance by seasonal flooding, combined with a cyclic high biomass input and massive microbial decomposition, inevitably influences the transformation and storage of nitrogen compounds in the soil.

### Dynamics of porewater nitrate

The concentration of soil porewater ${\text{NO}}_{3}^{-}$ in our study shifted dramatically from being undetectable in water-logged soil to $>1000\;\mu \text{mol}\;{\text{NO}}_{3}^{-}\;{\text{l}}^{-1}$ in drained soil indicating dynamic shifts between nitrate production and consumption in the soil. Extreme *in situ* concentrations of $10\text{ - 30mmol}\;{\text{NO}}_{3}^{-}\;{\text{l}}^{-1}$ observed in some samples might be ascribed to high evaporation and capillary forcing drawing nitrate-rich water up from deeper layers of soil and resulting condensation of nitrate near the surface (Wetselaar, 1960). At the beginning of the drained season at the sites of repeated sampling, we found that still water-logged soil contained no ${\text{NO}}_{3}^{-}$, but after 3–6 weeks of draining ${\text{NO}}_{3}^{-}$ could be found and increased further, presumably due to nitrification. This is supported by the O_2_ profiles (Figure 7) showing that drained soil was aerated to a depth of 20–50 cm interrupted only by short anoxic spells caused by a precipitation-induced increase in soil moisture.

Periodically flooded soils in the Amazon have similarly been found to be rich in inorganic nitrogen (Koschorreck, 2005). However, most of the inorganic nitrogen was removed during the first weeks of drying due to coupled nitrification-denitrification (Koschorreck, 2005), whereas decaying plant material may have supplied a continuous input of inorganic nitrogen to the Pantanal wetland soil.

As the wetland soil was draining at site A and B, only sparse plant growth (typically *Panicum maximum*) was observed, and 2–3 months after the end of the flooded season most of the soil surface at site A, B, and C was still covered with decaying aquatic macrophytes (largely *E. crassipes*). In the absence of inorganic nitrogen uptake by plant growth, we suggest that the large pool of nitrogen released from mineralization of the decaying plant residues was largely available for microbial nitrification and denitrification throughout the entire drained period. Such a continued input of nitrogen may explain the high nitrate content found in the Pantanal wetland soil even months after draining.

The water level during the 2009 flood was lower than average (Figure 2A) and areas that had not been flooded during the 2009 flooded season were covered by thick plant growth (e.g., *Costus spiralis, P. maximum*) by the end of the drained period in early 2010. In such non-flooded areas with high plant activity, plant-microbe competition for inorganic nitrogen would likely decrease the availability of inorganic nitrogen for soil microbial N transformations and gaseous N loss.

### N_2_O production and reduction processes in the soil

It is well known that sudden onset of anoxia (Bollmann and Conrad, 1998) and high concentrations of ${\text{NO}}_{3}^{-}$ (Blackmer and Bremner, 1978) can increase the emission of N_2_O. The four step reduction pathway of ${\text{NO}}_{3}^{-}$ to N_2_ is governed by four specific enzymes. The genes encoding each enzyme has multiple transcriptional promoters that are activated by different environmental parameters, and each enzyme has different substrate requirements and inhibitors (Zumft, 1997). This leads to temporary differences in the production and consumption rates of each intermediate causing a temporary accumulation of these intermediates (Frunzke and Zumft, 1986; Cervantes et al., 1998; Zhou et al., 2008). The high ${\text{NO}}_{3}^{-}$ content of the drained Pantanal wetland soil combined with sudden anoxia may have delayed or partly inhibited the reduction of N_2_O, stimulating a temporary high accumulation of N_2_O as seen in the experimentally flooded soil cores (Figures 8 and 9). The emitted N_2_O represented 35–38% of the registered cumulated net production (cumulated soil + emitted N_2_O, Figure 9) the rest being retained and reduced to N_2_ within the soil. Any additional N_2_O production balanced by simultaneous reduction to N_2_ would not be registered in this experimental set up and therefore it is unknown how much larger the N_2_ production was.

**Figure 10:**
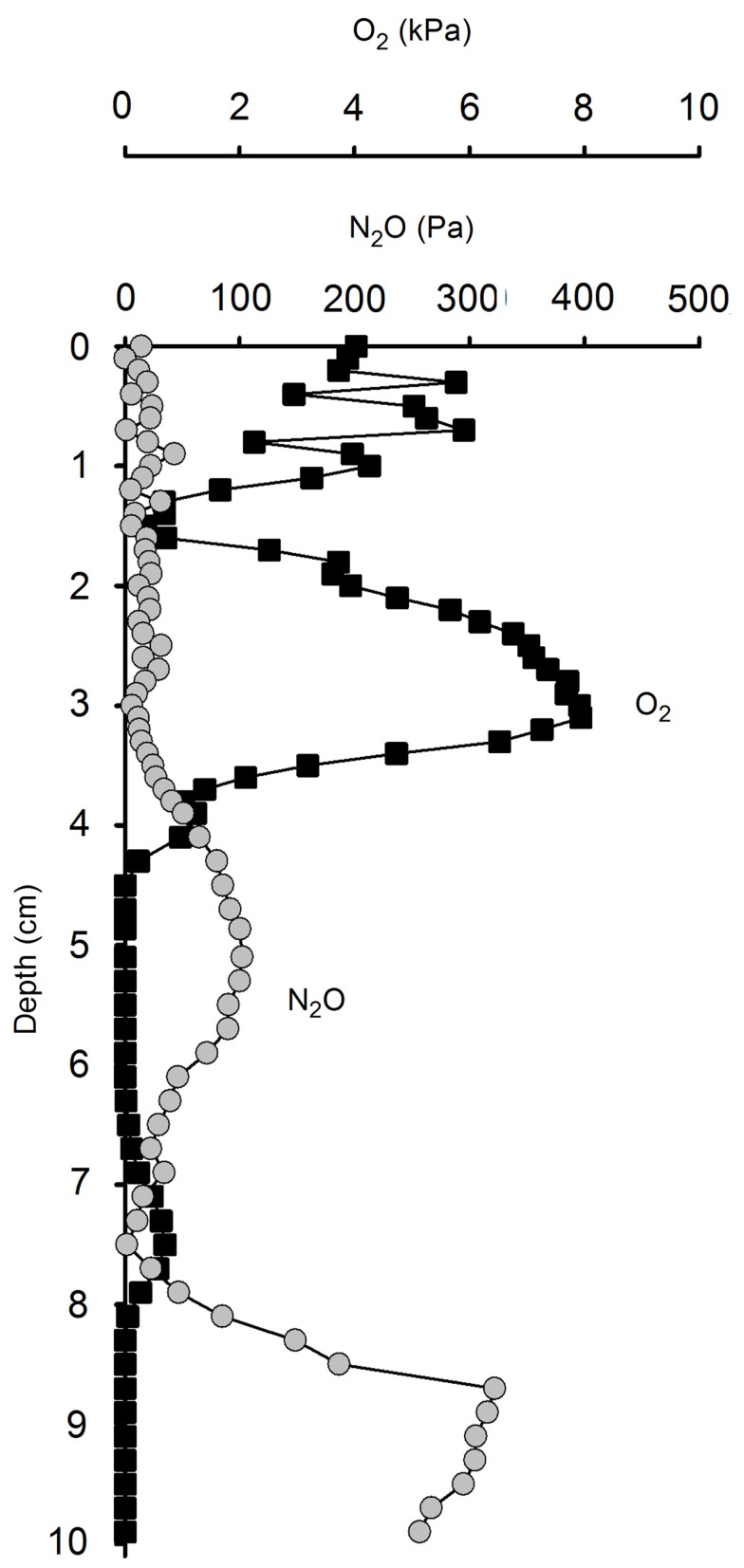
**Concentration profiles of N_2_O and O_2_ in a naturally re-wetted soil core**. The pores in this rain-wetted soil are partially gas-filled and partially water-filled. This creates steep gradients of N_2_O and O_2_ because of the much slower diffusion and lower solubility in water as compared to gas.

Microsensor measurements of O_2_ and N_2_O in soil cores sampled immediately after a natural, moderate rain event (Figure 10) revealed a heterogeneous distribution of O_2_ in the soil, reflecting a complex soil matrix with a combination of water-filled pores and gas-filled cracks, root channels, and other macro pores. During this partial water-logging of the soil the transport of gas was dominated by a mix of diffusion in water phase and gas phase, thereby allowing the fast escape of N_2_O produced several cm below the soil surface rather than being temporarily accumulated as a transient pool of N_2_O until being finally reduced to N_2_. Partial wetting of the soil by a moderate rain shower may thus favor hot moments (McClain et al., 2003) of shorter duration, but with higher N_2_O emissions than during complete waterlogging after heavy rain. Such a mixed situation of water-filled and gas-filled pore space has previously been associated with high N_2_O fluxes, even facilitating the efficient transport of N_2_O to the atmosphere (Markfoged et al., 2011).

At site A, we observed O_2_ penetration to a depth of 50–60 cm at Level 2, while oxidized iron was observed in soil layers at 80–90 cm depth in holes dug at Level 3. These observations, together with the observed increase in soil ${\text{NO}}_{3}^{-}$ content over time, suggest that nitrifiers could contribute to the production of N_2_O throughout the drained soil. Anaerobic microsites in the generally oxic layers may, however, cause locally intense denitrification (Smith, 1980). A relative increase of the anoxic volume in the soil, e.g., by temporary increased soil moisture as observed in the soil after a rain shower, would further favor denitrification temporarily.

Peak events of N_2_O flux from the drained Pantanal wetland soil were apparently closely coupled to precipitation events and variations in water content in the soil matrix (Figure 4). Increased soil moisture by a rain shower did not result in a complete depletion of O_2_ in the soil (Figure 7A), but rather increased the anoxic soil volume thus temporarily increasing the heterotrophic turnover of organic matter *via* denitrification. Fluctuations in the anoxic soil volume and fluctuating gas transport parameters thus, controlled the relative contribution of nitrification and denitrification to the total emission of N_2_O from the Pantanal wetland soil.

### Suggested N_2_O source strength of the Pantanal

As the drained wetland soil was rich in ${\text{NO}}_{3}^{-}$ at both the beginning and end of the drained season we suggest that the observed N_2_O emission continued throughout drained season. To calculate an estimate of the seasonal N_2_O flux we therefore classified each flux measurement as either a drained soil flux or a precipitation-triggered peak event flux. Considering the 10 sites as pseudo-replicates a total of 116 *in situ* flux measurements (each representing a mean of five chambers) were performed from 2008 to 2010. We classified 94 measurements as *drained soil fluxes* (mean = 0.43 ± 0.03 mmol N_2_O m^−2^ day^−1^), while 22 measurements were considered *precipitation-triggered peak events* (mean = 1.54 ± 0.24 mmol N_2_O m^−2^ day^−1^). The experimental flooding of soils (Figure 8) and the *in situ* N_2_O flux measurements (Figure 3) suggest that a typical peak event lasted ∼1 day. Therefore, cumulative emissions were calculated assuming linear changes between subsequent measurements of drained soil fluxes, while precipitation-triggered peak event fluxes were assumed to last 1 day.

Precipitation and soil moisture data at site A showed that, during 170 days of the drained season 2009–2010, there were at least six events of heavy precipitation and increased soil moisture that likely triggered a peak N_2_O emission event. We therefore assume that during the 170 day period precipitation-triggered peak events contributed 9.2 mmol N_2_O m^−2^, while non-wetted drained soil flux contributed 70.0 mmol N_2_O m^−2^ to the total emission of N_2_O. Consequently, we suggest that the cumulated N_2_O emission from the wetland soil during 170 days of the drained season was 79.3 mmol N_2_O m^−2^, with precipitation-triggered peak events contributing ∼12% of the total N_2_O emission. In contrast, wetting events in forest soil in Rondônia, Brazil were estimated by (Garcia-Montiel et al., 2003) to contribute <2% of the annual emissions.

For the purpose of estimating the N_2_O source strength of all the seasonally flooded soils in the Pantanal during the drained season, we calculated that the N_2_O flux from drained soil over a period of 170 days would be 0.30 Tg N (79.3 mmol N_2_O m^−2^ × 28 g N mol^−1^ × 1.4 × 10^11^ m^−2^ of seasonally flooded soil (Swarts, 2000). With an estimated global N_2_O source strength of 17.7 Tg N year^−1^ (IPCC, 2007) the Pantanal would thus contribute 1.7% to the global N_2_O emission budget, a significant single source of N_2_O.

Obviously, our calculations rely on extrapolation from a relatively small data set and need further confirmation by measurements over larger spatio-temporal scales. However, our findings are strongly supported by a recent analysis of the tropospheric distribution and variability of N_2_O which showed that N_2_O emissions are concentrated in the tropics and that South America has an up to five times higher emission of N_2_O than expected (Kort et al., 2011). In addition, the analysis by Kort et al. (2011) demonstrated that global N_2_O sources are concentrated in the tropics in November and January, thus coinciding with the drained season in the Pantanal and our findings of high N_2_O emission.

### Source of nitrogen

During 170 days of the drained season the loss of nitrogen from the soil *via* emission of N_2_O alone would be 158.5 mmol N m^−2^, requiring an annual input of at least 158.5 mmol N m^−2^ or 22.2 kg N ha^−1^ to balance this loss assuming that the system is in steady-state. So where does this nitrogen come from?

**Figure 11:**
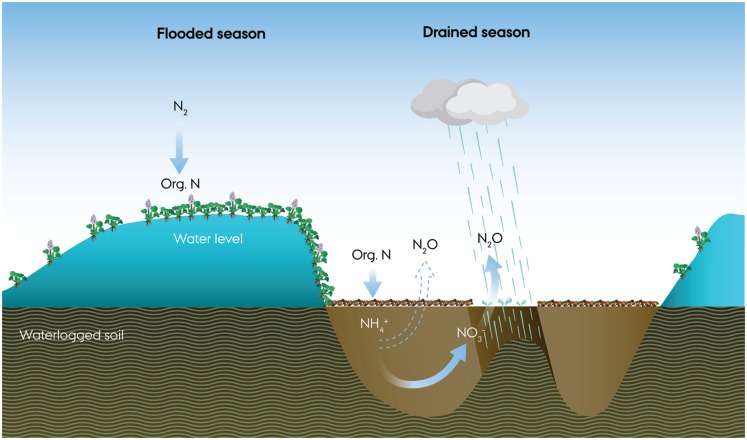
**A conceptual drawing of microbial nitrogen cycling and N_2_O emission during a 1 year flood cycle of the Pantanal**. During flooding, intense nitrogen fixation accompanies the growth of floating meadows dominated by water hyacinths (*Eichhornia crassipes*). As the water retreats, the dense, decaying mats release ammonium, and obscure light, preventing growth of other plants. As the soil is drained and aerated, O_2_ becomes available for intense nitrification in the soil while rain showers frequently deplete the O_2_ and elicit denitrification with bursts of N_2_O until the drained season ends with re-flooding of the soil.

Several reports indicate that natural tropical systems may export very large quantities of nitrogen (e.g., Martinelli et al., 1999; Matson et al., 1999), deemed the “*tropical nitrogen paradox*”, because input of nitrogen, presumably by N_2_-fixation, would have to occur in a nitrogen rich environment (Hedin et al., 2009). A spatial decoupling of the N_2_-fixation and the nitrogen rich soil due to epiphytic N_2_-fixers has been proposed to solve this paradox for tropical forest systems (Hedin et al., 2009). Could the seasonal production of aquatic macrophytes in the Pantanal be the natural nitrogen source driving nitrification and incomplete denitrification and N_2_O emission (Figure 11)?

Floating mats of *E. crassipes* have a seasonal biomass production of 10–20 t dry weight ha^−1^ with an estimated nitrogen content of 30–50 kg N t dry weight^−1^ and cover 5–100% of water bodies when the Pantanal is flooded (de Neiff et al., 2006). Such a decaying mat would supply the soil with 300–1,000 kg N ha^−1^ year^−1^ and ∼10 times as much carbon (Abdo and Da Silva, 2002; Xie et al., 2004), which eventually must be mineralized. As the water phase in the Pantanal was generally poor in inorganic nitrogen (Figure 6), we suggest that N_2_-fixing bacteria associated with the aquatic macrophytes (Iswaran et al., 1973; Purchase, 1977) are a major source of nitrogen to the system. Carignan and Neiff (1992) measured a total N_2_-fixation of 2.88 mmol N m^−2^ day^−1^ in a floating *E. crassipes* mat suggesting an input of 65–85 kg N ha^−1^ year^−1^. Our calculated loss of nitrogen of 22.2 kg N ha^−1^ from the Pantanal wetland soil *via* N_2_O would thus be in the range of 2–34% of the above estimated input of nitrogen to the soil *via* N_2_-fixation and aquatic macrophytes. This estimate is comparable to managed tropical soil where up to 28% of the applied N was lost as N_2_O (Veldkamp et al., 1998), but represents a much higher fraction than known from temperate soils. Fertilizer-induced N_2_O emission from temperate agricultural soils are generally in the range of 0.3–7% of the applied nitrogen fertilizer (Bouwman, 1996; Velthof et al., 2009), roughly a fivefold lower fraction than from the Pantanal wetland soil. The seasonal input of nitrogen by aquatic macrophytes, like the regular application of fertilizer, may therefore be the major cause of the high emission of N_2_O from the Pantanal wetland soil.

The carbon and nitrogen cycles are closely interlinked in wetlands and the large biomass input suggested above would imply a large input of carbon to the Pantanal wetlands. As the wetland soils of the Pantanal are not peat soils and have a C:N ratio of ∼10–20 (data not shown), this input of carbon must be mineralized. Evidence for such mineralization can be found in studies of CH_4_ production and emission from lakes and flooded areas during the flooded season (Marani and Alvala, 2007) and the low water period (Bastviken et al., 2010) suggesting an annual loss of CH_4_ to the atmosphere of 450–500 kg C ha^−1^ year^−1^, this also makes the Pantanal a significant source of the greenhouse gas CH_4_.

## Conclusion

Studies of nitrogen dynamics and N_2_O emissions from tropical freshwater wetlands are noticeably scarce. Furthermore, the contribution of N_2_O from tropical freshwater wetlands has largely been considered negligible (Matson and Vitousek, 1990). The six major tropical freshwater wetlands in South America are estimated to cover an area of 500,000 km^2^ that is flooded annually (Hamilton et al., 2002), while globally tropical wetlands are estimated to cover 5,000,000 km^2^ (Neue et al., 1997). Based on our measurements of the N_2_O flux from wetland soil we suggest that the Pantanal may be contributing 1.7% to the annual global N_2_O emission budget during the drained season; this is a significant hitherto ignored single source of N_2_O.

It is currently unknown to what extent the Pantanal wetland system with its dynamic cycling of nitrogen can be compared to other wetlands. The global N_2_O budget is not balanced (IPCC, 2007), which has been attributed to either a major unknown source or uncertainties in the quantification of one or more known sources (Smith, 1997). Kort et al. (2011) measured atmospheric concentrations of N_2_O suggesting that South America has a much higher emission of N_2_O than expected, supporting our observation of the Pantanal as a significant, but hitherto unknown source of N_2_O.

Our study underscores the direct and indirect importance of flooding and precipitation patterns in tropical watersheds and wetlands, where sudden natural wetting events can cause significant N_2_O emission comparable to heavily fertilized agricultural soils. This first study of the dynamics of soil nitrogen pools and emission of N_2_O from the world’s largest wetland thus emphasizes the current lack of knowledge about nitrogen cycling in undisturbed wetlands, and about how such systems may alter in response to a changing global climate. Lastly, it underscores the paramount importance of varying environmental boundary conditions modulating microbial mineralization processes in the carbon and nitrogen cycle of wetland soils.

## Conflict of Interest Statement

The authors declare that the research was conducted in the absence of any commercial or financial relationships that could be construed as a potential conflict of interest.
